# Soluble triggering receptor expressed on myeloid cell-1 reflects the cross-sectional activity of microscopic polyangiitis and granulomatosis with polyangiitis

**DOI:** 10.1016/j.heliyon.2023.e20881

**Published:** 2023-10-13

**Authors:** Taejun Yoon, Jang Woo Ha, Jung Yoon Pyo, Jason Jungsik Song, Yong-Beom Park, Sung Soo Ahn, Sang-Won Lee

**Affiliations:** a Department of Medical Science, BK21 Plus Project, Yonsei University, College of Medicine, Seoul, Republic of Korea; b Division of Rheumatology, Department of Internal Medicine, Yongin Severance Hospital, Yonsei University College of Medicine, Yongin, Gyeonggi-do, Republic of Korea; c Division of Rheumatology, Department of Internal Medicine, Yonsei University College of Medicine, Seoul, Republic of Korea; d Institute for Immunology and Immunological Diseases, Yonsei University College of Medicine, Seoul, Republic of Korea

**Keywords:** Microscopic polyangiitis, Granulomatosis with polyangiitis, Activity, Biomarker, sTREM-1

## Abstract

**Objectives:**

We investigated whether soluble triggering receptor expressed on myeloid cells-1 (sTREM-1) reflects cross-sectional activity of microscopic polyangiitis (MPA) and granulomatosis with polyangiitis (GPA).

**Methods:**

Forty-seven MPA and 32 GPA patients with well-documented clinical records and stored sera were enrolled. sTREM-1 levels were evaluated using Magnetic Luminex® assay, and disease activity was assessed using Birmingham vasculitis activity score (BVAS). Patients were divided into two groups according to the upper and lower halves of BVAS. Receiver operator characteristic (ROC) curve analysis was used to identify cut-off for determining upper half of BVAS. Linear and binary logistic regression was performed to evaluate the association between sTREM-1 and disease activity and status.

**Results:**

The median age of patients was 67.0 years, and 58.2 % were women. The median BVAS and sTREM-1 were 12.0 and 467.1 pg/mL. sTREM-1 was significantly correlated with BVAS along with five-factor score, Short-Form 36-Item Health Surveys, and C-reactive protein. In multivariable linear regression analysis, erythrocyte sedimentation rate (standardised β 0.241), and sTREM-1 (standardised β 0.288) were correlated with BVAS. ROC analysis revealed that the cut-off of sTREM-1 for the upper half of BVAS was 474.1 pg/mL. MPA and GPA patients with sTREM-1 ≥474.1 pg/mL exhibited a significantly higher risk for the upper half of BVAS than those without (relative risk 5.932). Multivariable logistic regression analysis demonstrated sTREM-1 ≥474.1 pg/mL (odds ratio 5.662) was associated with the upper half of BVAS.

**Conclusion:**

sTREM-1 reflects the activity of MPA and GPA, suggesting its role as a potential biomarker for assessing disease severity.

## Highlights

• Levels of serum sTREM-1 significantly correlated with disease activity in MPA/GPA.
• sTREM-1 levels were also shown to be useful in determining disease status in MPA/GPA.
• Measuring sTREM-1 levels could provide valuable information in patients with MPA/GPA.

## 1 Introduction

The triggering receptor expressed on myeloid cells-1 (TREM-1) is a transmembrane receptor primarily expressed on the surfaces of immune cells in the bloodstream such as neutrophils and monocytes [1]. In addition to lipopolysaccharide, TREM-1 may bind to diverse ligands for several receptors, including toll-like receptors (TLRs) or receptors for advanced glycation end products [2]. When pathogen-associated molecular patterns (PAMP) or damage-associated molecular patterns (DAMP) bind to TLRs, TLR-related signalling may induce innate immune responses and upregulate TREM-1 oligomerisation amplifying pro-inflammatory intracellular signals [3]. Meanwhile, as binding of ligand to an immunoglobulin-like domain of TREM-1 occurs, intracellular signal transduction can be initiated through the association between a cytoplasmic tail domain and adaptor molecules, in particular, DNAX activating protein of 12 kDa (DAP12) [3,4]. Ultimately, this intracellular signalling promotes the nuclear translocation of well-known inflammation-inducing transcription factors, resulting in augmented production of pro-inflammatory cytokines and chemokines [3]. The soluble TREM-1 (sTREM-1), which is a secreted immunoglobulin-like domain, can be produced in two ways: by either alternative splicing or cleavage of the extracellular domain of TREM-1 by matrix metalloproteinase [3,5]. Given that TREM-1-related intracellular pro-inflammatory signalling may enhance the expression of TREM-1 itself and MMP9, this could induce an increase in sTREM-1 production [6,7]. Therefore, sTREM-1 has the potential to reflect TREM-1-related activation and is considered as a biomarker of inflammatory burden [8].

Microscopic polyangiitis (MPA), and granulomatosis with polyangiitis (GPA), which are representative vasculitides of antineutrophil cytoplasmic antibody (ANCA)-associated vasculitis (AAV), are categorized as small-vessel vasculitides [9,10]. When primed neutrophils form a dimer with circulating ANCAs by both autoantigen-ANCA binding and Fc-gamma receptor-ANCA binding, they could be activated. Subsequently, these activated neutrophils can provoke inflammation in the vessel-adjacent tissues through migration, degranulation, radical oxygen production, and infiltration of effector immune cells into the corresponding tissues [11,12]. Additionally, complement 5a derived from the alternative complement pathway may aggravate organ injury [13]. Since pro-inflammatory cytokines and chemokines and inflammation-inducing transcription factors play key roles in the pathogenesis of MPA and GPA [14], TREM-1-related signalling could be inferred to reflect systemic inflammation; based on this theoretical background, several previous studies have demonstrated the clinical role of sTREM-1 in various autoimmune diseases including rheumatoid arthritis, systemic lupus erythematosus, and Behcet disease [[15], [16], [17]]. Additionally, a previous study showed that sTREM-1 was elevated in patients with active AAV compared to that in healthy participants [18]. Thus, sTREM-1 could be a useful biomarker of cross-sectional activity of MPA and GPA, which refers to the degree of disease severity at a specific time point. However, no studies have reported the potential of sTREM-1 in assessing activity MPA and GPA.

## 2 Materials and methods

### 2.1 Patient selection

We selected 79 patients with MPA and GPA from the Severance Hospital ANCA-associated VasculitidEs (SHAVE) cohort, which is a prospective observational cohort of AAV. A detailed description of this cohort is provided elsewhere [14]. All patients fulfilled the following inclusion criteria: 1) classified as MPA and GPA at the Division of Rheumatology in Severance hospital; 2) fulfilling the classification algorithm of AAV proposed by the European Medicine Agency in 2007 and the revised nomenclature of systemic vasculitides suggested by the Chapel Hill Consensus Conference in 2012 [9,10]. In addition, patients were also classified as MPA and GPA by applying the 2022 classification criteria for MPA and GPA proposed by the American College of Rheumatology and the European Alliance of Associations for Rheumatology retrospectively [19,20]; 3) available well-written medical records from which clinical, laboratory, radiological, and histological data and AAV activity, prognosis, and function related indices; 4) no concomitant serious medical conditions such as severe infectious diseases, malignancies and other systemic vasculitides mimicking MPA and GPA [21].

This study was approved by the Institutional Review Board of Severance Hospital, Seoul, Republic of Korea (**4**–**2022**–**1441**), and written informed consent was obtained from patients at initial enrolment in the SHAVE cohort.

### 2.2 Clinical and laboratory data collection

Age, sex, disease duration, disease remission status, AAV subtype, and ANCA positivity were recorded. Furthermore, the Birmingham vasculitis activity score (BVAS), five-factor score (FFS), Korean version of the Short-Form 36-Item Health Survey (SF-36) physical component summary (PCS), and mental component summary (MCS) were assessed for AAV activity, prognosis, and function related measures [[22], [23], [24]]. Myeloperoxidase (MPO)-ANCA and proteinase 3 (PR3)-ANCA were measured using an immunoassay with the novel anchor-coated, highly sensitive Phadia ELiA (Thermo Fisher Scientific/Phadia, Freiburg, Germany), and human native antigen using a Phadia 250 analyser, whereas perinuclear (P)-ANCA and cytoplasmic (C)-ANCA were detected using an indirect immunofluorescence assay. P-ANCA and C-ANCA were accepted equally as MPO-ANCA and PR3-ANCA [19,20]. Clinical systemic manifestations were assessed based on the items of BVAS, and the total score for each item was calculated. In addition, the presence and scoring of renal manifestation was evaluated according to the BVAS definitions that included: i) hypertension, ii) proteinuria >1+, iii) serum creatinine 125–249 μmol/L, iv) serum creatinine 250–499 μmol/L, v) serum creatinine ≥500 μmol/L, and vi) rise in serum creatinine >30 % or fall in creatinine clearance >25 % [22]. Results of acute-phase reactants of erythrocyte sedimentation rate (ESR) and C-reactive protein (CRP) were also collected.

### 2.3 Measurement of sTREM-1 in patients’ sera

Whole blood was collected from patients who provided consent and included in the SHAVE cohort, and serum was isolated from whole blood and stored at −80 °C prior to further analysis. Quantitative assessment of sTREM-1 in stored sera was done by the Human Magnetic Luminex® assay. Briefly, samples were diluted 1:2 with Calibrator Diluent RD6-52 and added to each well with Microparticle Cocktail. Then, incubation at room temperature was performed for 2 h. After 3 time washes, Biotin-Antibody Cocktail was added and incubated for 1 h at room temperature. Then, wells were washed 3 times and incubated with Streptavidin-PE for 30 min at room temperature. Final wash was performed and wells were read using a Luminex® MAGPIX instrument (Luminex, Austin, TX). A 5-parameter logistic curve was used with standard OD value for calculation.

### 2.4 Patient group according to BVAS and disease activity

We arbitrarily divided MPA and GPA patients into two groups according to BVAS ≥14 vs. BVAS <14, respectively, which was the upper and lower halves of BVAS. Meanwhile, disease remission was defined as: absence of disease activity attributable to active disease that requires continuous maintenance immunosuppressive therapy [25].

### 2.5 Statistical analyses

All statistical analyses were performed using IBM SPSS Statistics for Windows, version 26 (IBM Corp., Armonk, NY, USA). Continuous variables were expressed as medians with interquartile range, whereas categorical variables were expressed as numbers (percentages). The correlation coefficient (r) between the two continuous variables was obtained using the Pearson's correlation analysis. The standardised correlation coefficient (β) was obtained through the linear regression analysis, and significant differences between two continuous variables were compared using the Wilcoxon rank sum test. In addition, the optimal cut-off of sTREM-1 was extrapolated by the receiver operator characteristic (ROC) curve analysis, and the relative risk (RR) according to sTREM-1 level ≥474.1 pg/mL and 531.0 pg/mL was analysed using contingency tables and the chi-square test. The odds ratio (OR) was obtained using the multivariable logistic regression analysis, and for linear and logistic regression analyses, variables having statistical significance in the univariable analysis were included. In all statistical analysis, the significance was set as two-tailed P < 0.05.

## 3 Results

### 3.1 Characteristics of patients with MPA and GPA

The median age of the 79 patients (47 MPA and 32 GPA patients) was 67.0 years, and 58.2 % were women. The median BVAS, FFS, SF-36 PCS, and SF-36 MCS were 12.0, 2.0, 45.0, and 50.0, respectively, and 27.8 % of the patients were in remission. For systemic disease manifestations, the most frequent symptom was pulmonary manifestation (68.4 %), followed by renal manifestation (63.3 %). Total median scores of systemic items of BVAS calculated in pulmonary and renal manifestations were 4.0, and 6.0, respectively. The median ESR, CRP, and sTREM-1 were 55.0 mm/h, 8.0 mg/L, and 467.1 pg/mL, respectively (Table 1).

**Table 1:** Characteristics of patients with MPA and GPA (N = 79).

Variables	Values
Demographic data	
Age (years)	67.0 (54.0, 73.0)
Female gender (N, (%))	46 (58.2)
Disease duration (months)	0.0 (0.0–3.8)
Disease remission (N, (%))	22 (27.8)
***AAV subtype (N, (%))***	
MPA	47 (59.5)
GPA	32 (40.5)
***ANCA positivity (N, (%))***	
MPO-ANCA (or P-ANCA) positivity	58 (73.4)
PR3-ANCA (or C-ANCA) positivity	14 (17.7)
Both ANCA positivity	2 (2.5)
ANCA negativity	9 (11.4)
***AAV activity-, prognosis-, and function-related indices***	
BVAS	12.0 (7.0, 19.0)
FFS	2.0 (1.0, 2.0)
SF-36 PCS	45.0 (25.6, 61.9)
SF-36 MCS	50.0 (33.8, 65.2)
***Presence of systemic items of BVAS (N, (%)***	
General manifestation	31 (39.2)
Cutaneous manifestation	13 (16.5)
Mucous and ocular manifestation	3 (3.8)
Otorhinolaryngologic manifestation	27 (34.2)
Pulmonary manifestation	54 (68.4)
Cardiovascular manifestation	6 (7.6)
Gastrointestinal manifestation	1 (1.3)
Renal manifestation	50 (63.3)
Nervous systemic manifestation	21 (26.6)
***Total score of each systemic item of BVAS***	
General manifestation	0 (0, 2.0)
Cutaneous manifestation	0 (0, 0)
Mucous and ocular manifestation	0 (0, 0)
Otorhinolaryngologic manifestation	0 (0, 2.0)
Pulmonary manifestation	4.0 (0, 4.0)
Cardiovascular manifestation	0 (0, 0)
Gastrointestinal manifestation	0 (0, 0)
Renal manifestation	6.0 (0, 12.0)
Nervous systemic manifestation	0 (0, 1.0)
***Acute-phase reactants***	
ESR (mm/hr)	55.0 (15.0, 97.0)
CRP (mg/L)	8.0 (1.2, 65.7)
***sTREM-1 and cytokines (pg/mL)***	
sTREM-1	467.1 (344.2, 743.3)
TNF-α	7.1 (2.5, 11.3)
IL-6	8.8 (2.6, 21.8)

Values are expressed as a median (the first quartile, third quartile) or N (%).

MPA: microscopic polyangiitis; GPA: granulomatosis with polyangiitis; ANCA: antineutrophil cytoplasmic antibody; AAV: ANCA-associated vasculitis; MPO: myeloperoxidase; P: perinuclear; PR3: proteinase 3; C: cytoplasmic; BVAS: Birmingham vasculitis activity score; FFS: five-factor score; SF36: 36-item short form survey; PCS: physical component summary; MCS: mental component summary; WBC: white blood cell; BUN: blood urea nitrogen; ESR: erythrocyte sedimentation rate; CRP: C-reactive protein; sTREM-1: Soluble triggering receptor expressed on myeloid cells 1; TNF: tumour necrosis factor; IL: interleukin.

### 3.2 Correlation analysis of sTREM-1 with disease related indices

**Fig. 1:**
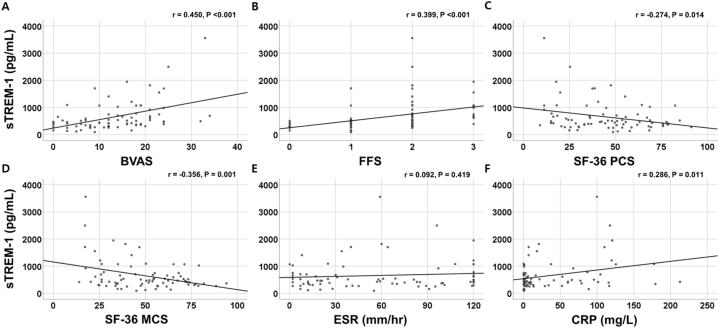
Correlation between sTREM-1 with disease related indices sTREM-1 was significantly correlated with BVAS (A), FFS (B), SF-36 PCS (C) and MCS (D), and CRP, but not ESR (E–F). The Pearson's correlation analysis was used to obtain the correlation coefficient (r) between the two continuous variables. sTREM-1: soluble triggering receptor expressed on myeloid cells-1; BVAS: Birmingham vasculitis activity score; FFS: five-factor score; SF-36: 36-item short form survey; PCS: physical component summary; MCS: mental component summary; CRP: C-reactive protein; ESR: erythrocyte sedimentation rate.

sTREM-1 showed a positive correlation with BVAS (r = 0.450, P < 0.001), FFS (r = 0.399, P < 0.001), and CRP (r = 0.286, P = 0.011). Conversely, sTREM-1 was inversely correlated with SF-36 PCS (r = −0.274, P = 0.014), and SF-36 MCS (r = −0.356, P = 0.001). However, sTREM-1 was not significantly correlated with ESR (r = 0.092, P = 0.419) (Fig. 1).

### 3.3 Linear regression analyses of variables with BVAS

Univariable linear regression analysis revealed that FFS, SF-36 PCS, SF-36 MCS, ESR, CRP, and sTREM-1 were significantly correlated with BVAS. Meanwhile, the multivariable analysis revealed that only ESR (standardised β = 0.241, 95 % confidence interval (CI) 0.001, 0.090, P = 0.044), and sTREM-1 (standardised β = 0.288, 95 % CI 0.001, 0.007, P = 0.010), were independently correlated with BVAS (Table 2).

**Table 2:** Linear regression analysis of variables for BVAS in patients with MPA and GPA.

Variables	Univariable			Multivariable		
	Beta	95 % CI	P value	Standardised Beta	95 % CI	P value
Demographic data						
Age (years)	0.146	−0.045, 0.213	0.200			
***AAV activity, prognosis, and function related indices***						
FFS	0.319	0.956, 4.921	0.004	0.153	−0.447, 3.260	0.135
SF-36 PCS	−0.482	−0.263, −0.110	<0.001	−0.231	−0.205, 0.026	0.128
SF-36 MCS	−0.434	−0.264, −0.095	<0.001	−0.190	−0.131, 0.116	0.902
***Acute-phase reactants***						
ESR (mm/hr)	0.419	0.040, 0.119	<0.001	0.241	0.001, 0.090	0.044
CRP (mg/L)	0.415	0034, 0.100	<0.001	0.032	−0.034, 0.045	0.798
***sTREM-1 (pg/mL)***	0.450	0.004, 0.009	<0.001	0.288	0.001, 0.007	0.010

The standardised correlation coefficient (β) was obtained by the linear regression analysis, and variables with statistical significance in the univariable analysis were included in the multivariable analysis.

BVAS: Birmingham vasculitis activity score; MPA: microscopic polyangiitis; GPA: granulomatosis with polyangiitis; AAV: ANCA-associated vasculitis; ANCA: antineutrophil cytoplasmic antibody; FFS: five-factor score; SF36: 36-item short form survey; PCS: physical component summary; MCS: mental component summary; ESR: erythrocyte sedimentation rate; CRP: C-reactive protein; sTREM-1: Soluble triggering receptor expressed on myeloid cells 1.

### 3.4 sTREM-1 levels according to BVAS cut-off and disease remission status

**Fig. 2:**
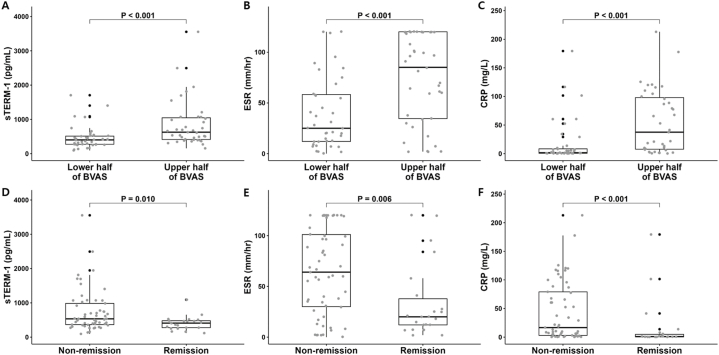
Difference of sTREM-1, ESR, and CRP according to BVAS cut-off and disease remission sTREM-1, ESR, and CRP levels in patients with the upper half of BVAS (A–C) and non-remission (D–F) were significantly higher than those in the lower half of BVAS and remission. Data are presented as medians and interquartile ranges, and individual data points are shown as jitters. Comparison of continuous variables was done by the Wilcoxon rank sum test. sTREM-1: soluble triggering receptor expressed on myeloid cells-1; ESR: erythrocyte sedimentation rate; C-reactive protein; BVAS: Birmingham vasculitis activity score.

Patients with the upper half of BVAS exhibited significantly higher median sTREM-1 (625.7 vs. 400.8 pg/mL), ESR (85.0 vs. 25.0 mm/h), and CRP (37.5 vs. 1.6 mg/L) than those with the lower half of BVAS (Fig. 2A). In addition, patients with remission state had significantly lower median sTREM-1 (410.0 vs. 534.5 pg/mL), ESR (20.0 vs. 64.0 mm/h), and CRP (1.1 vs. 16.7 mg/L) than those with non-remission state (Fig. 2B).

### 3.5 Derivation of optimal cut-off of sTREM-1 for the upper half of BVAS

**Fig. 3:**
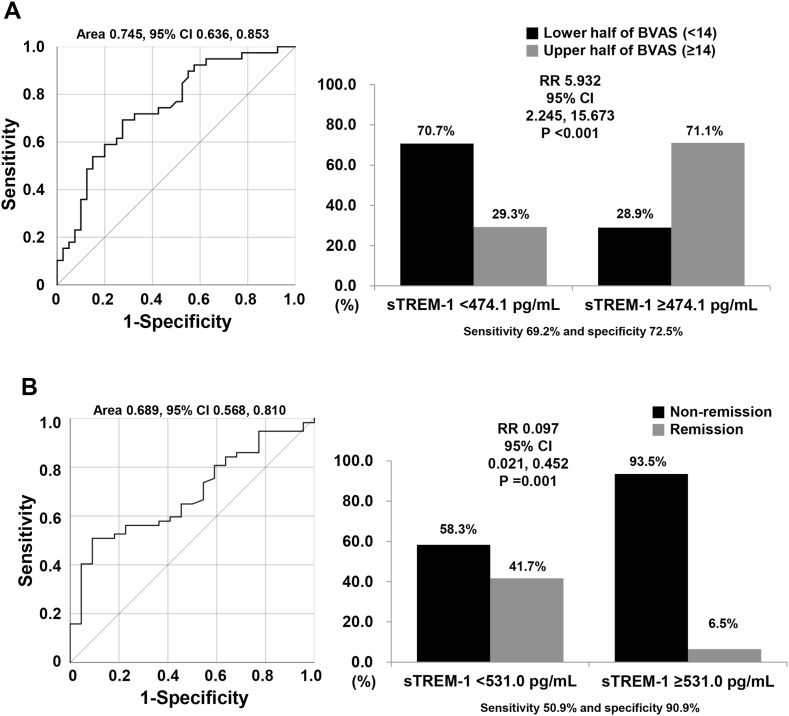
Optimal sTREM-1 cut-off and relative risk sTREM-1 of 474.1 pg/mL was set as the cut-off for the upper half of BVAS, and MPA and GPA patients with sTREM-1 ≥474.1 pg/mL exhibited a significantly higher risk for the upper half of BVAS than those without (A). For disease remission and non-remission, sTREM-1 cut-off 531.0 pg/mL showed the highest sensitivity and specificity, and those with sTREM-1 of ≥531.0 pg/mL had lower rate of being remission compared to those with sTREM-1 <531.0 pg/mL (B). The ideal cut-off of sTREM-1 was derived by the receiver operator characteristic curve analysis, and the relative risk was analysed using contingency tables and the chi-square test sTREM-1: soluble triggering receptor expressed on myeloid cells-1; BVAS: Birmingham vasculitis activity score; MPA: microscopic polyangiitis; GPA: granulomatosis with polyangiitis.

sTREM-1 had moderate performance (area under the curve [AUC] 0.745, 95 % CI 0.636, 0.853) to distinguish MPA and GPA patients with the upper half of BVAS, with a higher AUC compared to ESR (AUC 0.724, 95 % CI 0.608, 0.840) but lower than CRP (0.783, 95 % CI, 0.680, 0.886). Using the ROC curve of sTREM-1 for the upper half of BVAS, sTREM-1 of 474.1 pg/mL had the largest sum of sensitivity and specificity (sensitivity 69.2 %, specificity 72.5 %, positive likelihood ratio 2.5, negative likelihood ratio 0.2), and was set as the optimal cut-off for the upper half of BVAS (Fig. 3A).

### 3.6 Relative risk of according to sTREM-1 cut-offs

When we divided patients into two groups based on sTREM-1 ≥474.1 pg/mL, the upper half of BVAS was identified more frequently in patients with sTREM-1 ≥474.1 pg/mL than in those with sTREM-1 <474.1 pg/mL (71.1 % versus 29.3 %, P < 0.001). MPA and GPA patients with sTREM-1 ≥474.1 pg/mL exhibited a significantly higher risk for the upper half of BVAS than in those without (RR 5.932, 95 % CI 2.245, 15.673) (Fig. 3A).

On the other hand, in a ROC curve for discriminating remission and non-remission in these patients, sTREM-1 level 531.0 pg/mL was derived as an ideal cut-off value (AUC 0.689, 95 % CI 0.568, 0.810). Remission was less frequently found in those with sTREM-1 ≥531.0 pg/mL compared to patients with sTREM-1 <531.0 pg/mL (6.5 % versus 41.7 %, P = 0.001) Similarly, patients with sTREM-1 ≥531.0 pg/mL revealed a significantly lower rate of being in remission than in those without (RR 0.097, 95 % CI 0.021, 0.452) (Fig. 3B).

### 3.7 Logistic regression analyses of upper half of BVAS and high activity

In the univariable logistic regression analysis, FFS, SF-36 PCS, SF-36 MCS, ESR, and CRP were significantly associated with the upper half of BVAS. Additionally, both sTREM-1 and sTREM-1 ≥474.1 pg/mL were significantly associated with the upper half of BVAS. Continuous sTREM-1 or categorical sTREM-1 (sTREM-1 ≥474.1 pg/mL) was separately analysed in the multivariable logistic regression analyses. When sTREM-1 was included in the multivariable logistic regression analysis, FFS and ESR were independently associated with the upper half of BVAS; however, sTREM-1 showed no statistically significant association with the upper half of BVAS. However, when sTREM-1 ≥474.1 pg/mL was included in the multivariable analysis, sTREM-1 ≥474.1 pg/mL (OR 5.662, 95 % CI 1.346, 23.823, P = 0.018) was also independently associated with the upper half of BVAS, along with FFS (OR 2.280, 95 % 95 % CI 1.055, 4.930, P = 0.036) and ESR (OR 1.024, 95 % CI 1.005, 1.044, P = 0.012) (Table 3).

**Table 3:** Logistic regression analysis of variables for the upper half of BVAS in patients with MPA and GPA.

Variables	Univariable			Multivariable (sTREM-1)			Multivariable (sTREM-1 ≥ 474.1 pg/mL)		
	OR	95 % CI	P value	OR	95 % CI	P value	OR	95 % CI	P value
Demographic data									
Age (years)	1.031	0.996, 1.066	0.080						
***AAV activity, prognosis, and function related indices***									
FFS	3.117	1.643, 5.915	0.001	2.656	1.219, 5.788	0.014	2.280	1.055, 4.930	0.036
SF-36 PCS	0.965	0.943, 0.989	0.004	0.986	0.944, 1.030	0.531	0.993	0.949, 1.039	0.771
SF-36 MCS	0.969	0.945, 0.993	0.012	1.010	0.961, 1.061	0.707	1.007	0.959, 1.056	0.789
***Acute-phase reactants***									
ESR (mm/hr)	1.021	1.009, 1.033	0.001	1.021	1.004, 1.038	0.016	1.024	1.005, 1.044	0.012
CRP (mg/L)	1.018	1.006, 1.031	0.003	1.005	0.989, 1.021	0.535	1.008	0.991, 1.024	0.362
***sTREM-1 (pg/mL)***	1.002	1.001, 1.003	0.006	1.001	0.999, 1.003	0.164			
***sTREM-1 ≥ 474.1 pg/mL***	5.932	2.245, 15.673	<0.001				5.662	1.346, 23.823	0.018

The odds ratio (OR) was obtained through the multivariable logistic regression analysis, and for the multivariable analysis, variables with statistical significance in the univariable analysis were used.

BVAS: Birmingham vasculitis activity score; MPA: microscopic polyangiitis; GPA: granulomatosis with polyangiitis; AAV: ANCA-associated vasculitis; ANCA: antineutrophil cytoplasmic antibody; FFS: five-factor score; SF36: 36-item short form survey; PCS: physical component summary; MCS: mental component summary; ESR: erythrocyte sedimentation rate; CRP: C-reactive protein; sTREM-1: Soluble triggering receptor expressed on myeloid cells 1.

Since the arbitrary cut-off of BVAS of 14 could have influenced the results of our finding, an additional analysis was performed by defining high activity as the highest tertile of BVAS (BVAS ≥17). In this circumstance, sTREM-1 level of 601.2 pg/mL was found to be the optimal cut-off for high activity. In the univariable logistic regression analysis, FFS, SF-36 PCS, SF-36 MCS, ESR, CRP, sTREM-1, and sTREM-1 ≥ 601.2 pg/mL were significantly associated with high activity. In the multivariable analysis with sTREM-1, no variables were predictive of high disease activity; however, in a multivariable analysis including sTREM-1 ≥ 601.2 pg/mL as a covariate, only sTREM-1 ≥ 601.2 pg/mL (OR 5.526, 95 % CI 1.058, 28.872, P = 0.043) predicted with high activity (Supplementary Table 1).

### 3.8 sTREM-1 level and renal manifestation of MPA and GPA

Renal manifestation is one of the most life-threatening feature among patients with MPA and GPA [26]. On investigating the relationship between sTREM-1 and renal manifestation, sTREM-1 revealed to have significant correlation with its total score (r = 0.447, P < 0.001), and patients with renal manifestation exhibited significantly higher sTREM-1 than those without (median 639.7 vs. 344.2 pg/mL, P < 0.001). In addition, sTREM-1 demonstrated a correlation with both blood urea nitrogen (r = 0.705, P < 0.001) and serum creatinine (r = 0.783, P < 0.001), respectively (Supplementary Fig. 1).

## 4 Discussion

In this study, sTREM-1 levels were analysed in patients with MPA and GPA from a AAV cohort, and several interesting findings were observed. First, we found that sTREM-1 was significantly correlated with BVAS, FFS, SF-36 PCS, SF-36 MCS, and acute-phase reactant of CRP. In addition, a linear association with sTREM-1 and BVAS was demonstrated, and sTREM-1 level of over 474.1 pg/mL demonstrated that it was independently associated with the upper half of BVAS, which was identified similarly even when a definition of remission and different cut-off for BVAS was applied. Therefore, the results from this study provide evidence that sTREM-1 could have a potential as a biomarker to reflect the cross-sectional activity in patients with MPA and GPA.

The mechanism by which sTREM-1 is relevant to activity of MPA and GPA could be attributed to two different steps of disease pathogenesis and the role of sTREM-1 contributing in these processes [11,12]. The first step, which involves neutrophil priming and ANCA production, could be related to TREM-1 signalling that induces an overproduction of inflammatory cytokines [27]. This enables both neutrophil priming and cytoplasmic MPO and PR3 translocation to the cell surface and their extracellular release leading to an increase of circulating anti-MPO and anti-PR3. On the other hand, TREM-1-related signalling may also directly influence MPO production [28], which can augment the chance of producing autoantibodies against MPO. In addition, TREM-1 itself may increase the efficiency of autoantibody production, which was demonstrated using the inhibitor of TREM-1-related signalling in animal models of anti-GBM nephritis [29].

The next step, characterized by neutrophil activation and AAV development, could be reinforced by intracellular TREM-1-related signalling, mediated by membranous TREM-1 multimerisation stabilised by an adaptor molecule DAP12 [30]. This could lead to the promotion of neutrophil extravascular migration via AKT activation and NOX-2-dependent superoxide production [31], provoking inflammation in adjacent tissues of major organs. In addition, TREM-1-related signalling may accelerate radical oxygen species production [32], induce cell adhesion molecule-mediated neutrophil extracellular traps [33], and increase M1 macrophage polarization [34], which can form a vicious cycle of perpetuation inflammation in MPA and GPA. Therefore, it could be inferred that sTREM-1 may be associated with vasculitic activity in patients with MPA and GPA.

Among the systemic manifestation that was investigated, the two most common systemic items of BVAS were pulmonary and renal manifestations. However, while there was no correlation between sTREM-1 and total score of pulmonary manifestation, a significant association was demonstrated with total renal manifestation score, and patients with renal manifestation exhibited significantly higher sTREM-1 than those without. One possible reason for this phenomenon might be that TREM-1/sTREM-1 may have a higher association with organ-specific involvement in MPA and GPA, especially in the kidneys. Supporting this hypothesis, previous researches described the clinical role of TREM-1 in the development and aggravation of acute or chronic kidney diseases: TREM-1 was identified to participate in the pathogenesis of acute kidney disease using a TREM-1 inhibitor [35]; meanwhile, TREM-1 along with DAP12 was involved in macrophage polarization, leading to mediating tubular injury and interstitial fibrosis in obstructive nephropathy [36,37]. Moreover, TREM-1 was also induced and accelerated inflammatory renal injury in IgA nephropathy and an elevation of sTREM-1 was reported in patients with chronic kidney disease on haemodialysis [38,39]. These relationships between TREM-1 and sTREM-1 with kidney diseases might rationalize our results that sTREM-1 may predict the presence or extent of renal involvement of MPA and GPA. Nevertheless, because TREM-1 is widely expressed in myeloid cells and is also reported to contribute to lung injury [40,41], it remains to be clarified by additional studies whether there is a distinct role of TREM-1 and sTREM-1 in promoting organ-specific damage in inflammatory diseases.

The identification of sTREM-1 as a biomarker associated with disease activity in patients with MPA and GPA represents that measuring sTREM-1 hold promising implications for the clinical care in these population. Disease activity is a crucial factor influencing adverse clinical outcomes in MPA and GPA [42], however, laboratory markers that are clinically applicable in MPA and GPA is still lacking [43]. In this context, sTREM-1 could be a valuable tool for clinicians to monitor disease progression and response to treatment, which might aid in enhanced treatment decisions. Early identification of disease activity could have the potential of mitigating disease severity and reducing relapse rates through implementation of optimal therapeutic interventions.

## 5 Study Limitations

Several issues should be addressed as a limitation of this study. First, the number of patients with MPA and GPA was relatively small and a priori sample size was not calculated. Second, since only a single-point assessment of the relationship between soluble TREM-1 with disease activity in MPA and GPA was performed, changes of its levels during follow-up, after achieving remission, or according to therapeutic efficacy could not be evaluated, requiring further research. Third, because the SHAVE cohort is a mono-ethnic cohort of Korean patients, validation in other cohorts is necessary as the results of our study may be difficult to generalize. However, we believe that as a pilot study, this study is the first to clinically elucidate the role of sTREM-1 in MPA and GPA patients. Future studies with larger number of patients and additional clinical information could provide more reliable and dynamic information on the usefulness of sTREM-1 in assessing and monitoring AAV activity in real clinical settings.

## 6 Conclusion

To the best of our knowledge, this study is the first to demonstrate that sTREM-1 could be a potential biomarker reflecting the cross-sectional activity of MPA and GPA. Assessment of disease severity using sTREM-1 could be useful when making therapeutic decisions and have clinical implications in the management of MPA and GPA.

## Funding

This work was supported by the funding from CELLTRION PHARM, Inc. Chungcheongbuk-do, Republic of Korea (NCR 2019–6), and Chong Kun Dang Pharmaceutical Corp, Seoul, Republic of Korea. The funder was not involved in the study design, collection, analysis, interpretation of data, the writing of this article or the decision to submit it for publication.

## Ethics statement

This study was reviewed and approved by the Institutional Review Board of Severance Hospital, Seoul, Republic of Korea (**4**–**2022**–**1441**), and written informed consent was obtained from patients at initial enrolment in the SHAVE cohort.

## Data availability statement

Data associated with this study has not been deposited into a publicly available repository, but will be available from the corresponding author upon reasonable request.

## CRediT authorship contribution statement

**Taejun Yoon:** Conceptualization, Data curation, Formal analysis, Investigation, Methodology, Project administration, Validation, Visualization, Writing – original draft, Writing – review & editing. **Jang Woo Ha:** Conceptualization, Data curation, Formal analysis, Investigation, Methodology, Project administration, Software, Visualization, Writing – original draft, Writing – review & editing. **Jung Yoon Pyo:** Data curation, Investigation, Methodology, Project administration, Resources, Software, Writing – review & editing. **Jason Jungsik Song:** Investigation, Project administration, Resources, Software, Supervision, Writing – review & editing. **Yong-Beom Park:** Methodology, Resources, Software, Supervision, Writing – review & editing. **Sung Soo Ahn:** Conceptualization, Data curation, Formal analysis, Investigation, Methodology, Project administration, Resources, Software, Validation, Visualization, Writing – original draft, Writing – review & editing. **Sang-Won Lee:** Conceptualization, Data curation, Formal analysis, Funding acquisition, Investigation, Methodology, Project administration, Resources, Software, Supervision, Validation, Writing – original draft, Writing – review & editing.

## Declaration of competing interest

The authors declare the following financial interests/personal relationships which may be considered as potential competing interests:Sang-Won Lee reports financial support was provided by Chong Kun Dang Pharmaceutical Corp. Sang-Won Lee reports financial support was provided by CELLTRION PHARM.

## Abbreviations

AAV: antineutrophil cytoplasmic antibody-associated vasculitis
ANCA: antineutrophil cytoplasmic antibody
AUC: area under the curve
BVAS: Birmingham vasculitis activity score
C: cytoplasmic
CI: confidence interval
CRP: C-reactive protein
DAMP: damage-associated molecular patterns
DAP12: DNAX activating protein of 12 kDa
ESR: erythrocyte sedimentation rate
FFS: five-factor score
GPA: granulomatosis with polyangiitis
MCS: mental component summary
MPA: microscopic polyangiitis
MPO: myeloperoxidase
OR: odds ratio
P: perinuclear
PAMP: pathogen-associated molecular patterns
PCS: physical component summary
PR3: proteinase 3
ROC: receiver operator characteristic
RR: relative risk
SF-36: short-form 36-item health survey
SHAVE: Severance Hospital ANCA-associated VasculitidEs
sTREM-1: soluble triggering receptor expressed on myeloid cells-1
TLR: toll-like receptor
TREM-1: triggering receptor expressed on myeloid cells-1

## References

[1] Gao S., Yi Y., Xia G., Yu C., Ye C., Tu F., Shen L., Wang W., Hua C. (2019). The characteristics and pivotal roles of triggering receptor expressed on myeloid cells-1 in autoimmune diseases. Autoimmun. Rev..

[2] Siskind S., Brenner M., Wang P. (2022). TREM-1 modulation strategies for sepsis. Front. Immunol..

[3] Tammaro A., Derive M., Gibot S., Leemans J.C., Florquin S., Dessing M.C. (2017). TREM-1 and its potential ligands in non-infectious diseases: from biology to clinical perspectives. Pharmacol. Ther..

[4] Sun H., Feng J., Tang L. (2020). Function of TREM1 and TREM2 in liver-related diseases. Cells.

[5] Vandestienne M., Zhang Y., Santos-Zas I., Al-Rifai R., Joffre J., Giraud A., Laurans L., Esposito B., Pinet F., Bruneval P., Raffort J., Lareyre F., Vilar J., Boufenzer A., Guyonnet L., Guerin C., Clauser E., Silvestre J.S., Lang S., Soulat-Dufour L., Tedgui A., Mallat Z., Taleb S., Boissonnas A., Derive M., Chinetti G., Ait-Oufella H. (2021). TREM-1 orchestrates angiotensin II-induced monocyte trafficking and promotes experimental abdominal aortic aneurysm. J. Clin. Invest..

[6] Sun X.G., Duan H., Jing G., Wang G., Hou Y., Zhang M. (2019). Inhibition of TREM-1 attenuates early brain injury after subarachnoid hemorrhage via downregulation of p38MAPK/MMP-9 and preservation of ZO-1. Neuroscience.

[7] Weiss G., Lai C., Fife M.E., Grabiec A.M., Tildy B., Snelgrove R.J., Xin G., Lloyd C.M., Hussell T. (2017). Reversal of TREM-1 ectodomain shedding and improved bacterial clearance by intranasal metalloproteinase inhibitors. Mucosal Immunol..

[8] Jolly L., Carrasco K., Salcedo-Magguilli M., Garaud J.J., Lambden S., van der Poll T., Mebazaa A., Laterre P.F., Gibot S., Boufenzer A., Derive M. (2021). sTREM-1 is a specific biomarker of TREM-1 pathway activation. Cell. Mol. Immunol..

[9] Jennette J.C., Falk R.J., Bacon P.A., Basu N., Cid M.C., Ferrario F., Flores-Suarez L.F., Gross W.L., Guillevin L., Hagen E.C., Hoffman G.S., Jayne D.R., Kallenberg C.G., Lamprecht P., Langford C.A., Luqmani R.A., Mahr A.D., Matteson E.L., Merkel P.A., Ozen S., Pusey C.D., Rasmussen N., Rees A.J., Scott D.G., Specks U., Stone J.H., Takahashi K., Watts R.A. (2013). 2012 revised international Chapel Hill consensus conference nomenclature of vasculitides. Arthritis Rheum..

[10] Watts R., Lane S., Hanslik T., Hauser T., Hellmich B., Koldingsnes W., Mahr A., Segelmark M., Cohen-Tervaert J.W., Scott D. (2007). Development and validation of a consensus methodology for the classification of the ANCA-associated vasculitides and polyarteritis nodosa for epidemiological studies. Ann. Rheum. Dis..

[11] Choi C.B., Park Y.B., Lee S.W. (2019). Antineutrophil cytoplasmic antibody-associated vasculitis in Korea: a narrative review. Yonsei Med. J..

[12] Kitching A.R., Anders H.J., Basu N., Brouwer E., Gordon J., Jayne D.R., Kullman J., Lyons P.A., Merkel P.A., Savage C.O.S., Specks U., Kain R. (2020). ANCA-associated vasculitis. Nat. Rev. Dis. Prim..

[13] Jennette J.C., Falk R.J. (2014). Pathogenesis of antineutrophil cytoplasmic autoantibody-mediated disease. Nat. Rev. Rheumatol..

[14] Ahn S.S., Park Y.B., Lee S.W. (2021). Serological biomarkers and indices for the current activity and prognosis of ANCA-associated vasculitis: experience in a single centre in Korea. Yonsei Med. J..

[15] Choi S.T., Kang E.J., Ha Y.J., Song J.S. (2012). Levels of plasma-soluble triggering receptor expressed on myeloid cells-1 (sTREM-1) are correlated with disease activity in rheumatoid arthritis. J. Rheumatol..

[16] Bassyouni I.H., Fawzi S., Gheita T.A., Bassyouni R.H., Nasr A.S., El Bakry S.A., Afifi N. (2017). Clinical association of a soluble triggering receptor expressed on myeloid cells-1 (sTREM-1) in patients with systemic lupus erythematosus. Immunol. Invest..

[17] Jung Y.S., Kim S.W., Yoon J.Y., Lee J.H., Jeon S.M., Hong S.P., Kim T.I., Kim W.H., Cheon J.H. (2011). Expression of a soluble triggering receptor expressed on myeloid cells-1 (sTREM-1) correlates with clinical disease activity in intestinal Behcet's disease. Inflamm. Bowel Dis..

[18] Ajmani S., Singh H., Chaturvedi S., Mishra R., Rai M.K., Jain A., Misra D.P., Agarwal V. (2019). Utility of neutrophil CD64 and serum TREM-1 in distinguishing bacterial infection from disease flare in SLE and ANCA-associated vasculitis. Clin. Rheumatol..

[19] Robson J.C., Grayson P.C., Ponte C., Suppiah R., Craven A., Judge A., Khalid S., Hutchings A., Watts R.A., Merkel P.A., Luqmani R.A. (2022). 2022 American College of Rheumatology/European alliance of associations for Rheumatology classification criteria for granulomatosis with polyangiitis. Ann. Rheum. Dis..

[20] Suppiah R., Robson J.C., Grayson P.C., Ponte C., Craven A., Khalid S., Judge A., Hutchings A., Merkel P.A., Luqmani R.A., Watts R.A. (2022). 2022 American College of Rheumatology/European alliance of associations for Rheumatology classification criteria for microscopic polyangiitis. Ann. Rheum. Dis..

[21] Choi H., Shin E.C. (2021). Roles of type I and III interferons in COVID-19. Yonsei Med. J..

[22] Mukhtyar C., Lee R., Brown D., Carruthers D., Dasgupta B., Dubey S., Flossmann O., Hall C., Hollywood J., Jayne D., Jones R., Lanyon P., Muir A., Scott D., Young L., Luqmani R.A. (2009). Modification and validation of the Birmingham vasculitis activity score (version 3). Ann. Rheum. Dis..

[23] Guillevin L., Pagnoux C., Seror R., Mahr A., Mouthon L., Toumelin P.L. (2011). The Five-Factor Score revisited: assessment of prognoses of systemic necrotizing vasculitides based on the French Vasculitis Study Group (FVSG) cohort. Medicine (Baltim.).

[24] Han C.W., Lee E.J., Iwaya T., Kataoka H., Kohzuki M. (2004). Development of the Korean version of Short-Form 36-Item Health Survey: health related QOL of healthy elderly people and elderly patients in Korea. Tohoku J. Exp. Med..

[25] Mukhtyar C., Hellmich B., Jayne D., Flossmann O., Luqmani R. (2006). Remission in antineutrophil cytoplasmic antibody-associated systemic vasculitis. Clin. Exp. Rheumatol..

[26] Binda V., Moroni G., Messa P. (2018). ANCA-associated vasculitis with renal involvement. J. Nephrol..

[27] Roe K., Gibot S., Verma S. (2014). Triggering receptor expressed on myeloid cells-1 (TREM-1): a new player in antiviral immunity?. Front. Microbiol..

[28] Han L., Fu L., Peng Y., Zhang A. (2018). Triggering receptor expressed on myeloid cells-1 signaling: protective and pathogenic roles on streptococcal toxic-shock-like syndrome caused by Streptococcus suis. Front. Immunol..

[29] Du Y., Wu T., Zhou X.J., Davis L.S., Mohan C. (2016). Blockade of CD354 (TREM-1) ameliorates anti-GBM-induced nephritis. Inflammation.

[30] Carrasco K., Boufenzer A., Jolly L., Le Cordier H., Wang G., Heck A.J., Cerwenka A., Vinolo E., Nazabal A., Kriznik A., Launay P., Gibot S., Derive M. (2019). TREM-1 multimerization is essential for its activation on monocytes and neutrophils. Cell. Mol. Immunol..

[31] Baruah S., Murthy S., Keck K., Galvan I., Prichard A., Allen L.H., Farrelly M., Klesney-Tait J. (2019). TREM-1 regulates neutrophil chemotaxis by promoting NOX-dependent superoxide production. J. Leukoc. Biol..

[32] Arts R.J., Joosten L.A., van der Meer J.W., Netea M.G. (2013). TREM-1: intracellular signaling pathways and interaction with pattern recognition receptors. J. Leukoc. Biol..

[33] Murao A., Arif A., Brenner M., Denning N.L., Jin H., Takizawa S., Nicastro B., Wang P., Aziz M. (2020). Extracellular CIRP and TREM-1 axis promotes ICAM-1-Rho-mediated NETosis in sepsis. Faseb. J..

[34] Yang F.C., Chiu P.Y., Chen Y., Mak T.W., Chen N.J. (2019). TREM-1-dependent M1 macrophage polarization restores intestinal epithelium damaged by DSS-induced colitis by activating IL-22-producing innate lymphoid cells. J. Biomed. Sci..

[35] Siskind S., Royster W., Brenner M., Wang P. (2022). A novel eCIRP/TREM-1 pathway inhibitor attenuates acute kidney injury. Surgery.

[36] Lo T.H., Tseng K.Y., Tsao W.S., Yang C.Y., Hsieh S.L., Chiu A.W., Takai T., Mak T.W., Tarng D.C., Chen N.J. (2014). TREM-1 regulates macrophage polarization in ureteral obstruction. Kidney Int..

[37] Tammaro A., Stroo I., Rampanelli E., Blank F., Butter L.M., Claessen N., Takai T., Colonna M., Leemans J.C., Florquin S., Dessing M.C. (2013). Role of TREM1-DAP12 in renal inflammation during obstructive nephropathy. PLoS One.

[38] Zhao Y.F., Zhu L., Liu L.J., Shi S.F., Lv J.C., Zhang H. (2018). TREM-1 contributes to inflammation in IgA nephropathy. Kidney Dis..

[39] Essa E.S., Elzorkany K.M. (2015). sTREM-1 in patients with chronic kidney disease on hemodialysis. Apmis.

[40] Zhong W.J., Liu T., Yang H.H., Duan J.X., Yang J.T., Guan X.X., Xiong J.B., Zhang Y.F., Zhang C.Y., Zhou Y., Guan C.X. (2023). TREM-1 governs NLRP3 inflammasome activation of macrophages by firing up glycolysis in acute lung injury. Int. J. Biol. Sci..

[41] Liu T., Zhou Y., Li P., Duan J.X., Liu Y.P., Sun G.Y., Wan L., Dong L., Fang X., Jiang J.X., Guan C.X. (2016). Blocking triggering receptor expressed on myeloid cells-1 attenuates lipopolysaccharide-induced acute lung injury via inhibiting NLRP3 inflammasome activation. Sci. Rep..

[42] Solans-Laqué R., Rodriguez-Carballeira M., Rios-Blanco J.J., Fraile G., Sáez-Comet L., Martinez-Zapico A., Frutos B., Solanich X., Fonseca-Aizpuru E., Pasquau-Liaño F., Zamora M., Oristrell J., Fanlo P., Lopez-Dupla M., Abdilla M., García-Sánchez I., Sopeña B., Castillo M.J., Perales I., Callejas J.L. (2020). Comparison of the Birmingham vasculitis activity score and the five-factor score to assess survival in antineutrophil cytoplasmic antibody-associated vasculitis: a study of 550 patients from Spain (revas registry). Arthritis Care Res..

[43] Scurt F.G., Hirschfeld V., Schubert L., Mertens P.R., Chatzikyrkou C. (2023). Monitoring disease activity in antineutrophil antibody-associated vasculitis. Scand. J. Immunol..

