# Supplementary material for: Soluble triggering receptor expressed on myeloid cell-1 reflects the cross-sectional activity of microscopic polyangiitis and granulomatosis with polyangiitis
Source: Heliyon. 2023 Oct 13;9(10):e20881. doi: 10.1016/j.heliyon.2023.e20881 (PMC10597820; doi:10.1016/j.heliyon.2023.e20881)
Supplement: Supplementary file 1 [file mmc1.docx]

**Supplementary Table 1. Logistic regression analysis of variables for high activity of AAV (the highest tertile of BVAS*)* in patients with MPA and GPA**

| **Variables** | **Univariable** | | |  | **Multivariable**  **(sTREM-1)** | | |  | **Multivariable**  **(sTREM-1 ≥ 601.2 pg/mL)** | | |
| --- | --- | --- | --- | --- | --- | --- | --- | --- | --- | --- | --- |
|  | **OR** | **95% CI** | **P value** |  | **OR** | **95% CI** | **P value** |  | **OR** | **95% CI** | **P value** |
| ***Demographic data*** |  |  |  |  |  |  |  |  |  |  |  |
| Age (years) | 1.025 | 0.990, 1.063 | 0.165 |  |  |  |  |  |  |  |  |
| ***AAV activity, prognosis, and function related indices*** |  |  |  |  |  |  |  |  |  |  |  |
| FFS | 2.114 | 1.178, 3.793 | 0.012 |  | 1.686 | 0.815, 3.488 | 0.159 |  | 1.217 | 0.546, 2.714 | 0.631 |
| SF-36 PCS | 0.960 | 0.935, 0.985 | 0.002 |  | 0.991 | 0.949, 1.034 | 0.665 |  | 0.990 | 0.946, 1.035 | 0.653 |
| SF-36 MCS | 0.961 | 0.936, 0.987 | 0.004 |  | 1.000 | 0.954, 1.048 | 0.997 |  | 1.008 | 0.960, 1.058 | 0.758 |
| ***Acute-phase reactants*** |  |  |  |  |  |  |  |  |  |  |  |
| ESR (mm/hr) | 1.021 | 1.009, 1.033 | 0.001 |  | 1.013 | 0.997, 1.029 | 0.103 |  | 1.017 | 0.999, 1.035 | 0.059 |
| CRP (mg/L) | 1.022 | 1.010, 1.034 | <0.001 |  | 1.010 | 0.996, 1.025 | 0.163 |  | 1.012 | 0.997, 1.027 | 0.125 |
| ***sTREM-1 (pg/mL)*** | 1.001 | 1.000, 1.003 | 0.010 |  | 1.001 | 0.999, 1.002 | 0.229 |  |  |  |  |
| ***sTREM-1 ≥ 601.2 pg/mL*** | 5.100 | 1.872, 13.892 | 0.001 |  |  |  |  |  | 5.526 | 1.058, 28.872 | 0.043 |

The odds ratio (OR) was obtained using the multivariable logistic regression analysis, and multivariable analysis included variables with significance in univariable analysis.

BVAS: Birmingham vasculitis activity score; MPA: microscopic polyangiitis; GPA: granulomatosis with polyangiitis; AAV: ANCA-associated vasculitis; ANCA: antineutrophil cytoplasmic antibody; FFS: five-factor score; SF36: 36-item short form survey; PCS: physical component summary; MCS: mental component summary; ESR: erythrocyte sedimentation rate; CRP: C-reactive protein; sTREM-1: Soluble triggering receptor expressed on myeloid cells 1.
