# Supplementary figures and images for: Soluble triggering receptor expressed on myeloid cell-1 reflects the cross-sectional activity of microscopic polyangiitis and granulomatosis with polyangiitis
Source: Heliyon. 2023 Oct 13;9(10):e20881. doi: 10.1016/j.heliyon.2023.e20881 (PMC10597820; doi:10.1016/j.heliyon.2023.e20881)

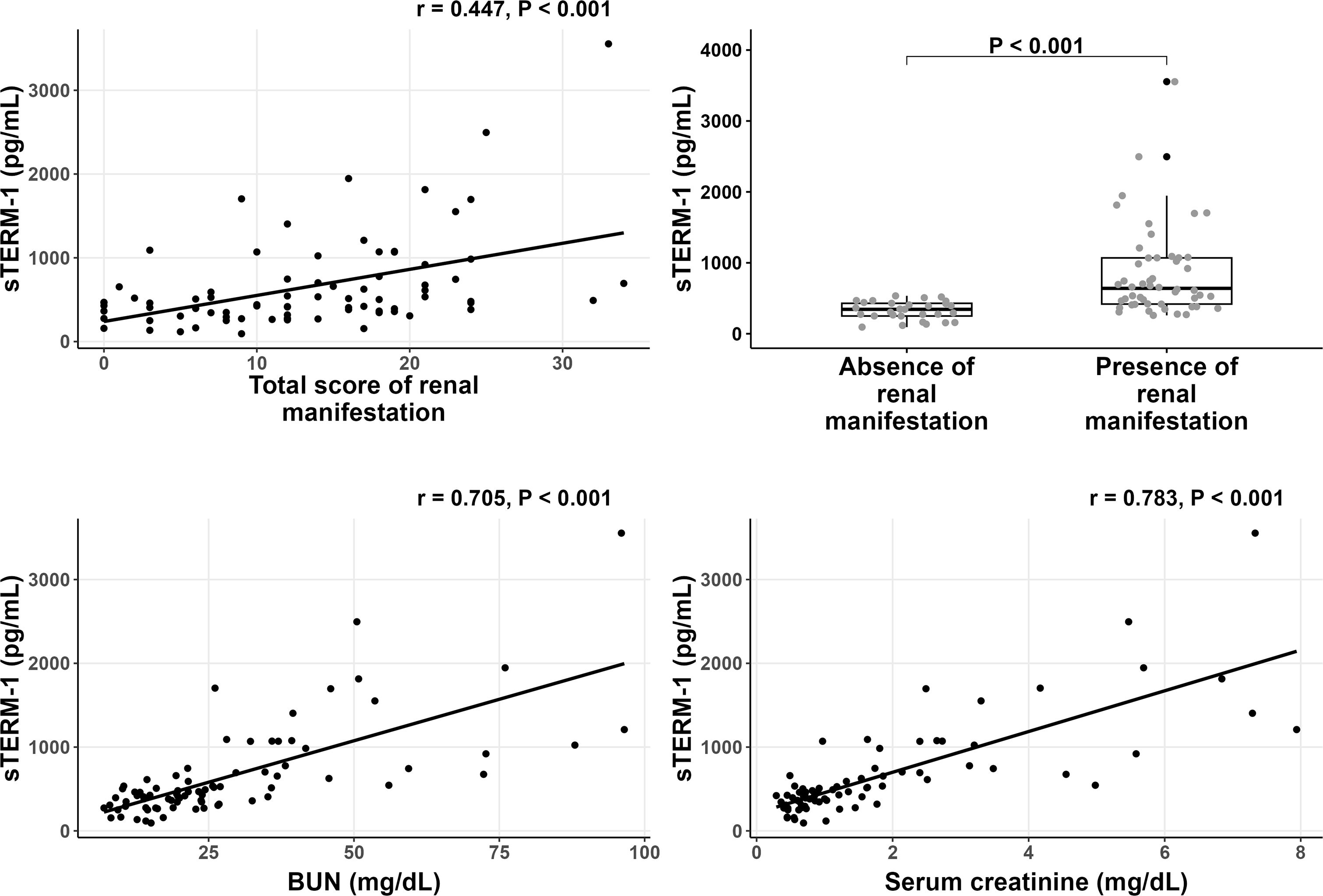

Supplement: Supplementary file 2 [file mmcfigs1.jpg]
